# Emotion Regulation, Eating Psychopathology, and Putative Transdiagnostic Psychological Processes: Findings from an Exploratory Network Analysis in a College Sample

**DOI:** 10.3390/nu16203452

**Published:** 2024-10-11

**Authors:** Tânia F. Rodrigues, Ricardo Silva, Fernando Fernández-Aranda, Paulo P. P. Machado

**Affiliations:** 1 Psychotherapy and Psychopathology Research Lab, Psychology Research Centre (CIPsi), School of Psychology, University of Minho, 4710-057 Braga, Portugal; 2 Psychological Neuroscience Laboratory, Psychology Research Centre (CIPsi), School of Psychology, University of Minho, 4710-057 Braga, Portugal; ricardoonssilva@gmail.com; 3 Clinical Psychology Department, University Hospital of Bellvitge, 08907 Barcelona, Spain; ffernandez@bellvitgehospital.cat; 4 CIBER Fisiopatología Obesidad y Nutrición (CIBERObn), Instituto de Salud Carlos III (ISCIII), 28029 Barcelona, Spain; 5 Psychoneurobiology of Eating and Addictive Behaviours Group, Neurosciences Programme, Bellvitge Biomedical Research Institute (IDIBELL), 08908 Barcelona, Spain; 6 Department of Clinical Sciences, School of Medicine and Health Sciences, University of Barcelona, 08007 Barcelona, Spain

**Keywords:** eating psychopathology, difficulties in emotion regulation, transdiagnostic psychological processes, interoceptive awareness, self-compassion, mindfulness, experiential avoidance, network analysis, prevention

## Abstract

Objective: Considering the prevalence of ED-related prodromal symptoms among higher education students (making them a population at risk for developing EDs), the main goals of this study were to conduct a network analysis in a college sample and to explore multivariate dependencies between a selection of empirically informed variables of interest to eating psychopathology, namely difficulties in emotion regulation and psychological processes (e.g., interoceptive awareness, self-compassion, self-criticism, mindfulness, and experiential avoidance). Methods: The sample included 294 college students (*M_age_* = 21.4, *SD* = 5.0; *M_BMI_* = 22.4, *SD* = 3.7). A Gaussian graphical network model was estimated to visualize interactions among the studied variables and to assess their centrality in terms of betweenness, closeness, strength, and expected influence. Results: A network system with 21 nodes was estimated (sparsity = 0.52). Nodes assessing disordered eating symptoms displayed the strongest correlation coefficients with nodes assessing dimensions of interoceptive awareness: eating concerns and not-distracting (*r* = −0.11), shape concerns and trusting (*r* = −0.16), and weight concerns and trusting (*r* = −0.10). Self-compassion was the node with the highest betweenness (SELFCS = 2.27) and closeness centrality (SELFCS = 1.70). The nodes with the highest strength centrality were strategies (DERS = 1.91) and shape concerns (EDE-Q = 1.51). Discussion: In this network model conducted in a college sample, eating-related symptoms were mainly associated with dimensions of interoceptive awareness. Also, the lack of effective strategies to regulate emotions, shape concerns, and self-compassion stood out as central nodes in the network model. The results suggest that addressing these variables may be promising in disrupting network systems marked by the presence of prodromal eating psychopathology symptoms in at-risk populations (e.g., college students).

## 1. Introduction

Eating disorders (EDs) are worldwide prevalent psychiatric conditions with typical onset in early adolescence [1]. The point prevalence of EDs was 3.06% in a Portuguese sample of female students in 2007 [2]. High prevalence, treatment relapse, and non-remission rates place EDs on a podium among one of the most challenging psychiatric disorders to achieve full remission [3]. Despite the wealth of targeted research over the past decades, the demand continues for etiological and maintenance models comprehensive enough to inform precision psychiatry in effectively addressing EDs.

One of the most consensual rationales across ED research posits the transdiagnostic role that difficulties in emotion regulation seem to play in the etiology and maintenance of EDs [4,5,6,7]. In a meta-analysis including studies in clinical samples, medium to large effect sizes were found for the associations between both adaptive and maladaptive emotion regulation strategies (particularly the lack of emotional awareness, clarity, and acceptance; reappraisal; problem-solving; rumination; avoidance; and suppression) and EDs [8]. In clinical samples including bariatric patients, the potential role of emotion regulation in surgery outcomes was also highlighted [9]. Also, Prefit et al. [8] highlighted studies conducted in non-clinical and college samples [10,11], in which ED-related symptoms were positively associated with a lack of emotional awareness, lack of clarity, and lack of emotional acceptance and negatively associated with goal-directed behaviors and cognitive reappraisal, with medium effect sizes. According to Breithaupt et al. [12], college students who exhibited eating disorder-related symptoms also displayed higher levels of maladaptive emotion regulation strategies such as rumination, experiential avoidance, and suppression in comparison to healthy controls (HC).

Adding to this, Monteleone and Cascino [13] conducted a systematic review of network analysis studies and found that both ED-specific symptoms (e.g., overvaluation of body shape and weight and desire to lose weight) and non-specific symptoms, also called symptoms from the “external field” (e.g., interpersonal difficulties, lack of interoceptive awareness, and emotion dysregulation-related problems), exhibited high centrality values in network models conducted in ED clinical samples [14,15]. The authors stressed the need to address relevant connections emerging between ED-specific symptoms and symptoms outside ED clusters, as the latter may contribute to the maintenance of the first ones [13]. Particularly, body checking, BMI, depression, and anxiety emerged as central variables in network models previously estimated in ED clinical samples [16,17]. Accordingly, Vervaet et al. [18] conducted a network study with a large ED clinical sample and found that perfectionism, maladaptive schemata, ineffectiveness, and interoceptive awareness were the most central nodes in the network, supporting their relevance as transdiagnostic vulnerability factors for eating psychopathology.

Interoceptive awareness refers to the metacognitive awareness of interoceptive cues and processes, at the conscious and unconscious level, through which internal sensory bodily stimuli are detected, translated, and integrated by the nervous system [19,20,21]. When impaired, it is considered to impact eating psychopathology via augmented difficulties in the interpretation of hunger/satiety bodily cues (hence resulting in problematic eating behaviors like overeating when already full or restricting food intake despite being hungry) [22] and via increased difficulties in discriminating body signals (e.g., hunger) from emotional states (e.g., anxiety), increasing the likelihood of failed attempts to regulate them [23,24]. In a previous study, Brown and colleagues [25] examined the factor structure of the multidimensional assessment of interoceptive awareness (MAIA) scale in a treatment-seeking transdiagnostic EDs sample and found that the subscales trusting, self-regulation, and emotional awareness significantly predicted ED symptoms and were negatively associated with difficulties in emotion regulation. Adding to this, Jenkinson and colleagues [26] found high levels of self-reported interoceptive deficits across EDs (including among recovered participants), suggesting that interoceptive awareness may be a transdiagnostic endophenotype in EDs and a potential target for prevention and treatment. Moment-by-moment interoceptive processing of stimuli generated in biological systems (e.g., cardiovascular, visceral, and gastrointestinal) enables the adaptive selection and implementation of goal-directed actions aimed at maintaining homeostatic regulation [21,27]. Even though excessive attentional focus toward physical sensations is associated with psychopathology (e.g., panic disorder), contemplative-based awareness of our internal sensory landscape, instead of anxiety-driven attentional focus, is fundamental to effectively regulate emotions [21,28]. On the other hand, contextual-based psychological processes (e.g., experiential acceptance, mindfulness, self-compassion, etc.) seem to buffer the pathogenic effect of difficulties in emotion regulation and eating psychopathology, particularly via one’s ability to approach suffering or discomfort (e.g., unpleasant/undesired internal experiences like visceral/physical sensations) with acceptance and self-compassion, instead of avoidance and self-criticism [29,30,31].

According to dimensional taxonomic systems of psychopathology (e.g., network approaches), psychiatric disorders result from complex and dynamic interactions between symptoms, in which each symptom (or node) interacts with another, either activating or de-activating and strengthening or weakening the network [14]. In network studies, nodes can be assessed in terms of their relative centrality to the network system—nodes with the highest statistical centrality are theoretically inferred as prominent to the network model, potentially contributing to the symptomatic landscape of the sample under study [18,32,33]. Moreover, network systems show dynamic and distinct presentations over time. Borsboom [14] proposed four distinct phases in the establishment of networks: (1) an initial asymptomatic phase, in which the network system components display weak associations with each other; (2) the surge of an external component that triggers interactions among latent components in the network system; (3) the start of dynamic feedback loops between specific network components; and (4) the establishment of a self-sustained network system of symptoms that remains active even after the initial trigger becomes absent—a phenomenon referred to as hysteresis [34].

Since networks assessing ED symptoms may—as the phenomenon of hysteresis suggests—display distinct phenomenological features across time [15,34], it remains relevant to explore both prodromal and established ED symptoms in non-clinical and clinical samples [35,36]. For instance, Stice and colleagues [37] published a paper clarifying the sequencing of symptom emergence in EDs and found that attitudinal symptoms like weight and shape overvaluation began as prodromal symptoms that predicted the later onset of EDs. The authors emphasized the need for prevention programs focused on ED prodromal symptoms, especially in at-risk populations. Also, Kambanis et al. [38] examined the longitudinal symptomatic course observed in participants diagnosed with avoidant/restrictive food intake disorder (ARFID), who presented ED diagnostic crossover (to restricting and binge-eating/purging spectrums). Among these individuals, ED symptoms—namely shape and weight concerns—preceded the adoption of disordered eating behaviors. According to the authors, these findings point to the relevance of tackling early shape and weight concerns in order to prevent symptomatic exacerbation and ED onset. However, despite the ones mentioned above, additional exploratory studies are warranted to further establish which psychological variables stand out as relevant transdiagnostic processes for eating psychopathology throughout its developing continuum, including prodromal stages.

Considering the prevalence of ED-related prodromal symptoms among higher education students (making them a population at risk for developing EDs), the main goals of this study were to conduct a network analysis in a college sample and to explore the combined interaction of an empirically driven selection of variables of interest to eating psychopathology, namely difficulties in emotion regulation and transdiagnostic adaptive (dimensions of interoceptive awareness, self-compassion, and mindfulness) and maladaptive psychological processes (experiential avoidance, self-criticism, and experiential avoidance). We expected positive associations between ED-related symptoms and difficulties in emotion regulation and maladaptive psychological processes and negative associations with adaptive psychological processes. Also, we hypothesized that, in a college sample with unestablished/subthreshold eating psychopathology levels, nodes with the highest centrality in the network will reflect critical prodromal symptoms worthy of consideration in EDs prevention programs.

## 2. Methods

### 2.1. Participants

The sample included 294 participants with ages comprehended between 18 and 56 years old (*M* = 21.4, *SD* = 5.0). A total of 25 (8.5%) were male participants, 267 (90.8%) were female participants, and 2 (0.7%) did not report. Participants’ BMI varied between 15.1 and 51.0 (*M* = 22.4, *SD* = 3.7). Most participants were Portuguese (*n* = 271; 92.2%), single (*n* = 280; 95.2%), had a secondary education level (*n* = 196; 66.7%), and were Psychology students/working students enrolled in a Portuguese higher education institution. A total of 29 (9.9%) participants reported a current/past history of ED (e.g., anorexia nervosa [AN], bulimia nervosa [BN], binge eating disorder [BED], or unknown), and 75 (25.5%) were taking regular medication (e.g., contraceptive pills, antihistamines, antidepressants, etc.).

### 2.2. Measures

Participants in this study completed an initial questionnaire developed to collect self-reported sociodemographic and clinical data (e.g., age, sex, occupation, education level, weight, height, etc.) and the Portuguese version of the following measures:

The eating disorder examination questionnaire (EDE-Q) [39,40], which comprises 22 continuous items rated on a 7-point Likert scale (0–6) that assess core ED symptoms and 6 items to assess the self-reported frequency of disordered eating behaviors over the past 28 days. A global score and four subscale scores (restraint, eating concern, shape concern, and weight concern) are generated.

The difficulties in emotion regulation scale (DERS) [41,42], which comprises 36 continuous items rated on a 5-point Likert scale (1–5) that assess levels of difficulties in emotion regulation. A total score and six subscale scores (strategies, nonacceptance, awareness, impulse, goals, and clarity) are generated.

The multidimensional assessment of interoceptive awareness-version 2 (MAIA-2) [21], which comprises 37 items rated on a 6-point Likert scale (0–5) to assess self-reported dimensions of interoceptive awareness. The Portuguese version—MAIA-P—with 33 items and seven factors (noticing, not-distracting, not-worrying, attention regulation, emotional awareness, self-regulation, and trusting) was used [43].

The acceptance and action questionnaire-II (AAQ-II) [44,45], which comprises 7 continuous items rated on a 7-point Likert scale (1–7), generating a total score of experiential avoidance.

The mindful attention awareness scale (MAAS) [46,47], which comprises 15 items rated on a 6-point Likert scale (1–6), generating a total score to assess dispositional mindfulness.

The self-compassion scale (SELFCS) [48,49], which comprises 26 items rated on a 5-point Likert scale (1–5) that assess self-compassion facets and its opposites. A total score and six subscale scores (self-kindness, self-judgment, common humanity, isolation, mindfulness, and over-identification) are generated.

The forms of self-criticizing/attacking and self-reassuring scale (FSCRS) [50,51], which comprises 22 items rated on a 5-point Likert scale (0–4) to assess self-criticism and self-reassurance. Three subscale scores (inadequate self, hated self, and reassured self) are generated. A composite score of self-criticism is calculated by the sum of the scores of inadequate self and hated self.

### 2.3. Procedures

The participants in this sample were recruited from a Portuguese higher education institution through an institutional research platform. The students were presented with a briefing on the study. The inclusion criteria in this study were being a college student and being able to provide informed consent. The exclusion criteria included being currently pregnant and being under 18 years old. Only participants fulfilling the criteria to participate were granted access to the study. Seven participants did not go through with their participation and were excluded from the final dataset. After being informed of the voluntary, anonymous, and confidential nature of the study, those willing to participate completed the assessment. The completers were assigned curricular credits as compensation for their participation. This study was approved by the institution’s Ethics Committee board, in line with the Declaration of Helsinki.

### 2.4. Statistical Analysis

The software IBM^®^ SPSS Statistics (28.0.0.0, 190; IBM Corp.©, Armonk, NY, USA, 2021) was used to perform exploratory and descriptive statistical analyses. No missing values were observed in the dataset. Univariate outliers were identified and excluded through the inspection of *z*-score values (<3.29 or >3.29). Values of skewness and kurtosis were considered to examine the response distribution of the data (*Sk* < 3; *Ku* < 10) [52]. Descriptive statistics of the variables under study were calculated to characterize the sample. The internal consistency of the variables was calculated.

**Figure 1:**
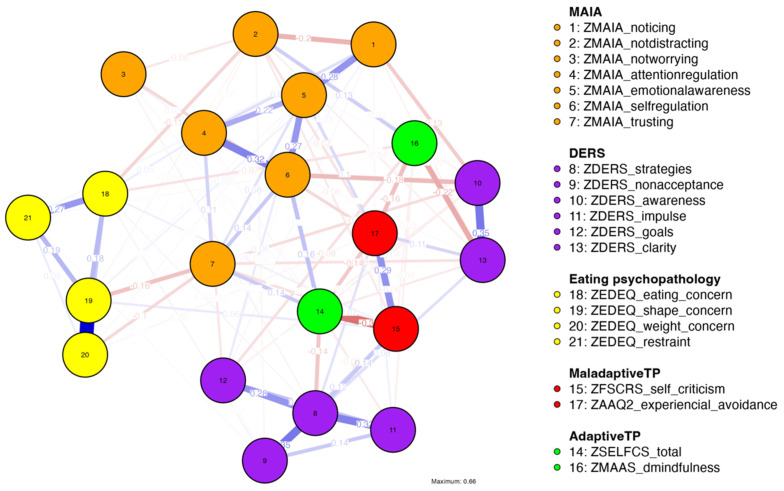
Network model (Gaussian graphical model) displaying relationships between eating psychopathology (EDE-Q), difficulties in emotion regulation (DERS), dimensions of interoceptive awareness (MAIA), self-criticism (FSCRS), experiential avoidance (AAQ-II), self-compassion (SELFCS), and mindfulness (MAAS). Nodes represent observed variables. Nodes’ colors indicate cluster membership. Edges represent raw partial correlation coefficients between two nodes (after controlling for the effect of the remaining variables in the model). Blue lines represent positive associations, and red lines represent negative associations. Thicker lines indicate stronger correlation coefficients. The absence of edges between nodes indicates non-significant associations after partial control of the effect of the remaining variables in the model.

The software JASP (version 0.19) [53] was used to run a network analysis using the bootnet R package [54], which allows the visualization of nodes (observed variables) and the interactions between them (edges) and the estimation of the strength of these interactions (edge-weights), considering multivariate covariances of the data. A Gaussian graphical model (GGM) network was run including a pool of variables theoretically interrelated, resulting in an observable network structure organized according to the strength of associations between the variables of interest (Figure 1). The method ‘Extended Bayesian Information Criterion Graphical Least Absolute Shrinkage and Selection Operator’ (EBICglasso) was selected to estimate the network model. This method takes under consideration the number of parameters to be estimated and the limited *N* of the sample (i.e., it accounts for model fit and model complexity) by applying a regularization penalty and limiting the number of parameters estimated in order to deal with multiplicity issues (e.g., type I errors) [54,55]. The ‘Auto’ correlation method, suitable for continuous variables (producing Person’s *r* coefficients), was selected.

**Figure 2:**
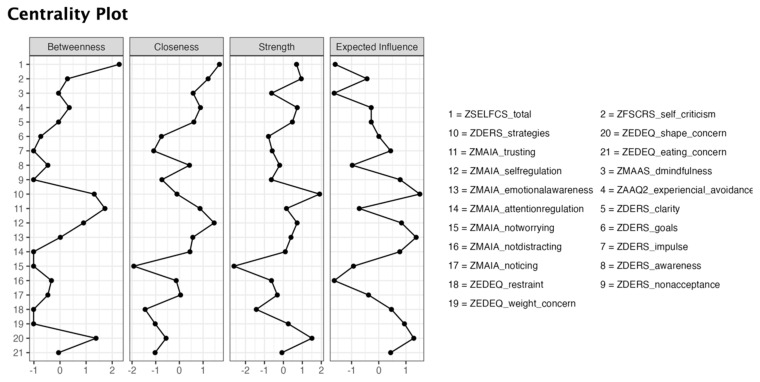
Centrality indices. Higher betweenness, closeness, strength, and expected influence values indicate greater centrality of a node. Values are standardized.

To explore the relative importance of the nodes in the network, a series of centrality indices were considered (Figure 2): betweenness—the number of shortest paths passing through a focal node; closeness—the inverse of the sum of the shortest path lengths of a focal node; strength—the sum of the absolute values of the edge weights of a focal node; and expected influence—an index of centrality proxy to strength in which negative edges are also accounted for [56].

**Figure 3:**
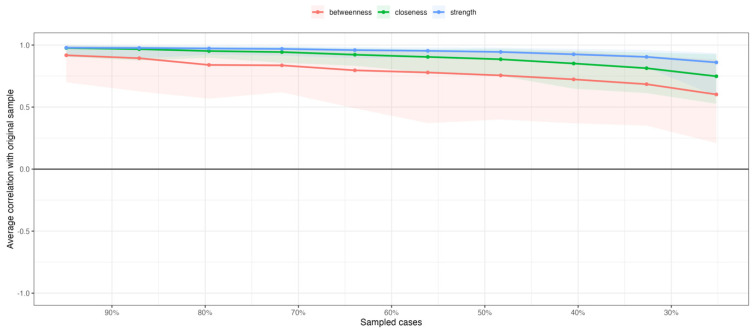
Averaged correlation coefficients between the centrality indices estimates of the original sample and the centrality indices estimates of the resampled subsets (random case-dropping without replacement). Lines represent means. Centrality indices are expected to remain stable after case-dropping.

**Figure 4:**
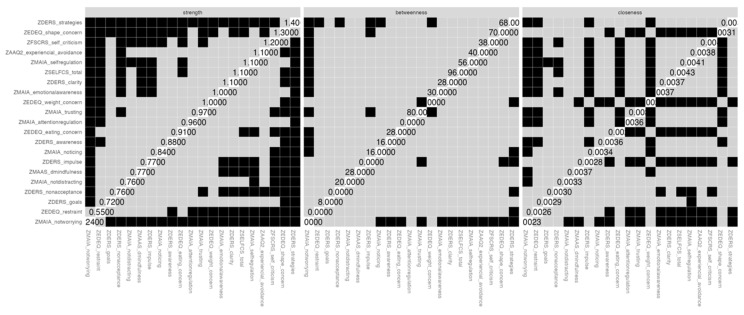
Bootstrapped difference tests for the centrality indices. Black boxes represent significant differences between nodes. Grey boxes represent non-significant differences.

A series of tests were considered to examine the stability of the indices of centrality and the accuracy of the edge weights [56]. Case-dropping subset bootstrap was used to investigate the stability of the centrality indices, in which subsets of the data are re-estimated with fewer cases (randomly dropped). Similar magnitudes of association between the original sample and the resampled subsets are expected (Figure 3). A non-parametric bootstrap technique was used to examine statistically significant differences between nodes’ centrality estimates (Figure 4). To assess the accuracy of edge weights, a nonparametric bootstrapping technique set to 1000 resamples was used to examine bootstrapped confidence intervals (CI). Larger CIs indicate less accurate edge weights. Bootstrapped CIs of resampled cases with replacement were plotted (Supplementary Figure S1).

## 3. Results

### 3.1. Preliminary and Descriptive Statistics and Internal Consistency

Based on the examination of the z-scores, 11 outliers were removed. The coefficients of skewness and kurtosis were acceptable (*Sk* < 3; *Ku* < 10). The means, standard deviations, minimum/maximum values, and Cronbach’s alpha values for the variables under study are presented in Table 1.

### 3.2. Network Analysis

#### 3.2.1. Network Estimation Parameters

The estimated GGM network had a total of 21 nodes, 100 non-zero edges (of 210 possibilities), and a sparsity value of 0.52.

The analysis of the partial correlation coefficients and the visual inspection of the network model (Figure 1) indicated that nodes assessing eating psychopathology (EDE-Q) displayed the strongest partial correlation coefficients with nodes assessing dimensions of interoceptive awareness (MAIA), particularly between eating concerns (EDE-Q) and not-distracting (MAIA; *r* = −0.11), shape concerns (EDE-Q) and trusting (MAIA; *r* = −0.16), and weight concerns (EDE-Q) and trusting (MAIA; *r* = −0.10).

Overall, the strongest associations in the entire network were displayed between nodes within the same cluster: shape concerns and weight concerns (EDE-Q; *r* = 0.66), awareness and clarity (DERS; *r* = 0.35), and strategies and non-acceptance (DERS; *r* = 0.35). With regards to the associations between nodes from different clusters, the strongest associations were between self-compassion (SELFCS) and self-criticism (FSCRS; *r* = −0.37), self-criticism (FSCRS) and experiential avoidance (AAQ-II; *r* = 0.29), and clarity (DERS) and mindfulness (MAAS; *r* = −0.22).

To examine the accuracy of edge weights, bootstrapped CIs are displayed in Supplementary Figure S1.

#### 3.2.2. Centrality Indices

Regarding the estimates of the centrality indices (Figure 2), the nodes with the highest betweenness centrality were self-compassion (SELFCS_betweeness_ = 2.27), trusting (MAIA_betweeness_ = 1.72), shape concerns (EDE-Q _betweenness_ = 1.38), and strategies (DERS_betweeness_ = 1.31). The items with the highest closeness centrality were self-compassion (SELFCS_closeness_ = 1.70), self-regulation (MAIA_closeness_ = 1.47), and self-criticism (FSCRS_closeness_ = 1.21). The items with the highest strength centrality were strategies (DERS_strenght_ = 1.91) and shape concerns (EDE-Q _strenght_ = 1.51). Finally, the items with the highest expected influence (EI) centrality were strategies (DERS_EI_ = 1.51), emotional awareness (MAIA_EI_ = 1.38), and shape concerns (EDE-Q_EI_ = 1.29).

#### 3.2.3. Centrality Stability

Figure 3 plots the case-dropping bootstrap analysis used to confirm the stability of the centrality indices, as data cases are randomly dropped without replacement. The X-axis shows the correlation coefficients between the original sample (with 100% of the cases) and the resampled subsets. The visual inspection of the plot indicates that strength, followed by closeness, are the most stable centrality indices, holding magnitudes of association above 0.70 between the original sample and the resampled subsets. The betweenness centrality index trends toward weaker magnitudes of association with the original sample’s estimates, as the resampled subsets’ size decreases.

Figure 4 depicts the centrality difference test for the strength index. Lack of adaptive emotion regulation strategies (DERS, strategies), body shape concerns (EDE-Q, shape concern), self-criticism (FSCRS), self-regulation (MAIA), and self-compassion (SELFCS) were the nodes that most significantly differed from the remaining.

## 4. Discussion

In this study, a network approach was used to explore multivariate dependencies between nodes assessing eating psychopathology, difficulties in emotion regulation, and transdiagnostic psychological mechanisms (dimensions of interoceptive awareness, self-compassion, self-criticism, mindfulness, and experiential avoidance) in a sample of college students. Research using psychopathological networks has gained momentum during the last years in the field of clinical psychology [54]. To the extent of our knowledge, this is the first study considering the interplay between this selection of empirically informed variables in a network analysis.

In the network model, the strongest partial correlation coefficients in measures of eating psychopathology (eating, shape, and weight concerns) were observed in relation to dimensions of interoceptive awareness, namely not-distracting and trusting. These results align with previous studies emphasizing the role of interoceptive awareness in ED clinical samples [13,18,57] and the predictive role of self-regulation, not-distracting and trusting in detecting ED symptoms [25]. According to Kaye [57], monitoring and processing interoceptive cues (e.g., temperature, muscular sensations, vasomotor flush, etc.) is critical to maintaining body homeostasis and integrating cognitive and affective processes in the body, which is crucial for effective self-regulation. Previously, Brown et al. [25] observed how ED-diagnosed individuals who display lower scores on dimensions of interoceptive awareness may present biased integration of interoceptive cues and increased interoceptive prediction errors–discrepancies between expected and experienced bodily signals [25]. Such interplay may compromise one’s ability to identify emotion- and eating-related interoceptive cues or to distinguish them, leaving little opportunity to select adaptive emotion regulation strategies when necessary.

The estimation of the centrality indices allowed the identification of the most central variables in the network [58]. In this study’s network model, the nodes with the highest betweenness centrality were self-compassion (SELFCS), trusting (MAIA), shape concerns (EDE-Q), and strategies (DERS). Higher betweenness values indicate that a node behaves as a bridge between several nodes in a network, i.e., it is located in a proximal crossing path between other pairs of nodes [59]. The nodes with the highest closeness centrality were self-compassion (SELFCS), self-regulation (MAIA), and self-criticism (FSCRS), and the nodes with the highest strength centrality were strategies (DERS) and shape concerns (DERS). Finally, the nodes with higher values of expected influence were strategies (DERS), shape concerns (DERS), and emotional awareness (MAIA). Higher values generally indicate that a node is more likely to affect other nodes/symptoms in the network [59].

Interestingly, the observed results suggest a noteworthy centrality of ED-related symptoms (particularly shape concerns), hindered interoceptive awareness, and limited access to effective emotion regulation strategies, especially considering the non-clinical nature of the sample. Concerning dimensions of interoceptive awareness, trusting—the subjective experience of sensing one’s body as safe—, self-regulation—the ability to regulate distress via awareness of bodily sensations—, and emotional awareness—one’s awareness of the association between bodily sensations and emotional states—, achieved particular relevance in the network model. Also, the prominent place of self-compassion in the network model in terms of its degree of betweenness and closeness is worthy of mentioning, indicating that this transdiagnostic process variable frequently fell in the shortest path between other pairs of nodes in the model.

According to Depue [60], at least three types of emotion regulation systems can be distinguished in humans. The threat and protection system is linked to mammalian defenses, triggered in the face of threats or evolutionary disadvantages, and associated with psychopathology when under- or over-developed. The drive system is resource-focused and linked to our motivational systems to seek pleasure, pursue goals, compete, and leverage social status. Finally, the soothing system is related to the absence of perceived threats, to the subjective perception of contentment and safety and to predisposition towards social connectedness ([61] p. 200). According to Gilbert [61], when the soothing system is active, individuals feel safe and connected and experience positive emotions. The practice of self-compassion favors the activation of the soothing system, and individuals with higher levels of self-compassion are less likely to over-rely on the threat and drive systems to meet ends or self-regulate in the face of challenges. Although self-compassion is not substantially connected to ED nodes in the model, the inspection of the network plot reveals positive edges emerging between self-compassion and interoceptive awareness (trusting and self-regulation), while the strongest negative edges emerge between self-compassion and self-criticism, experiential avoidance, and the strategies dimension of the DERS. This may indicate that those participants displaying higher levels of self-compassion may, even in the face of challenging internal or external landscapes, process internal cues accurately, regulate physiological and emotional distress, and feel safe in their own bodies throughout the process (compatible with higher levels of trusting and self-regulation). On the other hand, individuals less prone to accommodate personal suffering and discomfort in a contemplative, warm, and supporting way may resort to defensive and anxiety-driven strategies to self-regulate (as suggested by the negative associations with self-criticism, experiential avoidance, and lack of effective strategies to regulate emotions). These findings align with studies in ED clinical samples, highlighting the joint relevance of experiential avoidance, self-criticism [62,63], and self-compassion [30] for eating psychopathology. In line with previous reports documenting the relevance of self-compassion in the treatment of EDs [30], our results further support the association between self-compassion, difficulties in emotion regulation, interoceptive awareness, and ED attitudinal symptoms in a college sample.

Finally, and worthy to mention, unexpected positive associations (although negligible) were found between ED variables and some dimensions of interoceptive awareness (noticing, attention regulation, and emotional awareness). We hypothesize that the extent to which dimensions of interoceptive awareness remain negatively associated with ED symptoms may be tied to confounding variables. For instance, individuals with lower levels of dispositional mindfulness may not be able to override noisy pathogenic attention-related processes (e.g., attention bias towards negative stimuli). In these cases, one’s attentional focus on interoceptive cues may become anxiety-driven [21,28], potentially increasing difficulties in emotion regulation and ED-related symptoms. Future studies are warranted to explore the nature of these associations and putative moderating variables.

### 4.1. Clinical Implications

According to Epskamp et al. [54], multivariate dependencies estimated in network models may inform exploratory hypothesis generation in psychopathology network systems. Since associations between nodes with statistical relevance may indicate potential causal effects [14], these results may hold important implications for EDs prevention.

Previous reports showed that ED symptoms like eating and weight concerns significantly predicted the later onset of EDs [37], emphasizing the relevance of prevention programs able to tackle prodromal ED symptoms in at-risk populations. Current findings partially support the well-established notion that college students may be at increased risk of developing full or subthreshold EDs [2], particularly given the salience of nodes assessing shape concerns in this network model.

Also, when considering the development of EDs preventive programs, it may be worth considering the psychological process variables emerging as central in network models, along with ED-specific symptoms. This idea was put forward by Monteleone and Cascino [13], who stressed the importance of considering the “external field” in the treatment of EDs. Thus, the results in this study bring attention to the relevance of the dimensions of interoceptive awareness to the sustainability of the network structure, hence reinforcing the potential of including bottom-up processing-related strategies in the therapeutic process that bring focus to the mind–body integration of sensory states as a fundamental intermediate step to successfully acquire emotion-regulation skills [20,64]. In line, Olatunji and colleagues [65] conducted a network study in which interoceptive awareness and infectiveness were central EDs symptoms that predicted treatment outcome. Also, Khoury and colleagues [20] reviewed a series of studies in which targeting interoception improved ED treatment outcomes. For instance, cognitive behavioral therapy (CBT) with interoceptive exposure was effective in increasing interoceptive awareness in patients diagnosed with bulimia nervosa [66] and binge eating disorder [67]. Psychotherapeutic approaches that rely mainly on top-down processing strategies and typically focus on cognition reframing or behavioral modification (e.g., CBT) are valuable pathways for self-regulation via executive functions [68]. In addition, therapeutic components that enhance mindful awareness and acceptance of internal experiences without judgment (e.g., mindfulness- and acceptance-based interventions) may boost the enhancement of interoceptive awareness.

Borsboom [14] argued how precision psychiatry may optimize therapeutic gains by adapting treatment modalities to the patients’ heterogeneous needs. However, as noted by Levinson and collaborators [33], it is not yet empirically established whether intervening in central nodes in a network system produces significant changes in the remaining ones. Thus, future studies are needed to explore the actual impact of adding empirically-based adjunctive treatment components destined to tackle idiosyncratic vulnerability psychological processes (e.g., limited ability to feel safe in one’s body and predisposition to avoid undesired internal experiences), if and when they become clinically prominent.

Overall, the results of this study point to the putative potential of fostering emotion regulation skills, interoceptive awareness skills (trusting, self-regulation, not-distracting, and emotional awareness), and self-compassion among individuals presenting ED-related symptoms at subthreshold/prodromal levels.

### 4.2. Limitations and Future Directions

Some limitations must be considered in this study. This is a sample consisting of higher education students. Future studies in clinical samples are warranted before generalizing these findings to individuals diagnosed with an ED. Nevertheless, this study may be elucidative to understanding prodromal ED presentations.

The number of variables used in the network model may pose limitations to the estimation of its parameters. To try and circumvent this, the EBICglasso method was selected to estimate the model, as it applies a penalty term for model fit and complexity, and bootstrapped CIs were used to examine the accuracy of edge weights and centrality stability.

Moreover, in this study, less than 10% of the sample was formed by male participants, and the participants presented a wide spectrum of ages. Since the reduced sample size compromised the estimation of the network model across age and gender subgroups, future studies with larger samples could consider gender and age variability, considering the existing research indicating differences across genders regarding emotion regulation abilities [69]. Our sample also included participants with a wide BMI range. Considering previous accounts suggesting a putative influence of BMI on interoceptive skills [70], present results must be considered with caution, and future studies are needed to explore the confounding effect of BMI in the relationship between ED-related symptoms and interoceptive awareness.

Another limitation is related to the use of self-report measures that rely on participants’ accuracy and may be influenced by social desirability. This may be particularly problematic when it comes to the study of interoception, since self-report may be biased due to a lack of self-awareness [26]. Future studies may bypass this limitation through multi-method assessment protocols able to evaluate psychophysiological correlates of interoceptive awareness. Also, future studies could consider the use of instruments that measure meta-cognitive emotion regulation strategies (e.g., cognitive reappraisal), considering their relevance in relation to eating psychopathology in clinical and non-clinical samples [8].

Although in network studies partial correlations constitute potential causal effects [14], the results in this study are exploratory, and the hypotheses generated do not dispense future studies including idiographic research methods and time series analysis to assess the prospective stability of edge weights and centrality indices.

## 5. Conclusions

Network studies may enhance our current understanding of psychopathology and the related protective/vulnerability transdiagnostic mechanisms under a transdiagnostic umbrella. In this network model conducted in a college sample, eating-related symptoms were mainly associated with specific dimensions of interoceptive awareness. Also, the lack of effective strategies to regulate emotions, shape concerns, and self-compassion stood out as central nodes in the network model. The results suggest that addressing these variables may be promising in disrupting network systems marked by the presence of prodromal eating psychopathology symptoms in at-risk populations (e.g., college students).

## Figures and Tables

**Table 1:** Descriptive statistics and internal consistency of the variables under study.

	Total Sample (*N* = 294)		
	*M (SD)*	Min–Max	*α*
EDE-Q			
Eating concern	0.5 (0.8)	0–3.8	0.78
Shape concern	1.7 (1.5)	0–5.9	0.93
Weight concern	1.4 (1.4)	0–5.8	0.85
Restraint	0.8 (1.1)	0–5.0	0.84
Global score	1.1 (1.1)	0–4.7	0.95
MAIA			
Noticing	2.8 (1.2)	0–5.0	0.70
Not-distracting	2.9 (1.3)	0–5.0	0.90
Not-worrying	2.5 (0.9)	0–4.8	0.57
Attention regulation	2.4 (1.1)	0–5.0	0.90
Emotional awareness	3.2 (1.1)	0–5.0	0.87
Self-regulation	2.2 (1.0)	0–5.0	0.89
Trusting	2.9 (1.3)	0–5.0	0.89
DERS			
Strategies	17.5 (7.1)	8–40	0.91
Nonacceptance	12.9 (6.5)	6–30	0.95
Impulse	12.4 (5.3)	6–30	0.90
Goals	14.2 (4.7)	5–25	0.90
Clarity	11.6 (4.1)	5–24	0.83
Awareness	14.5 (4.5)	6–27	0.83
Total score	83.1 (25.0)	36–159	0.96
SELFCS			
Total score	19.1 (4.5)	7.7–29.3	0.95
FSCRS			
Self-criticism	18.3 (12.4)	0–56	0.94
AAQ-II			
Experiential avoidance	23.6 (9.7)	7–49	0.92
MAAS			
Dispositional mindfulness	4.0 (1.0)	1.1–6.0	0.92

Note: EDE-Q—eating disorder examination questionnaire; MAIA—multidimensional assessment of interoceptive awareness; DERS—difficulties in emotion regulation scale; SELFCS—delf-compassion scale; FSCRS—forms of self-criticizing/attacking and self-reassuring scale; AAQ-II—acceptance and action questionnaire-II; MAAS—mindful attention awareness scale.

## Data Availability

The data that support this study’s findings are not publicly available due to ethical and legal restrictions and are available upon reasonable request to the corresponding author.

## Supplementary Materials

The following supporting information can be downloaded at: https://www.mdpi.com/article/10.3390/nu16203452/s1, Figure S1: *Bootstrapped* confidence intervals (CIs) of edge-weights of resampled cases with replacement.
