# Supplementary material for: Emotion Regulation, Eating Psychopathology, and Putative Transdiagnostic Psychological Processes: Findings from an Exploratory Network Analysis in a College Sample
Source: Nutrients. 2024 Oct 11;16(20):3452. doi: 10.3390/nu16203452 (PMC11510149; doi:10.3390/nu16203452)

Supplementary Figure S1. *Bootstrapped* confidence intervals (CIs) of edge-weights of resampled cases with replacement. Red and thicker line corresponds to the sample values. Horizontal gray lines correspond to edge-weights confidence intervals.

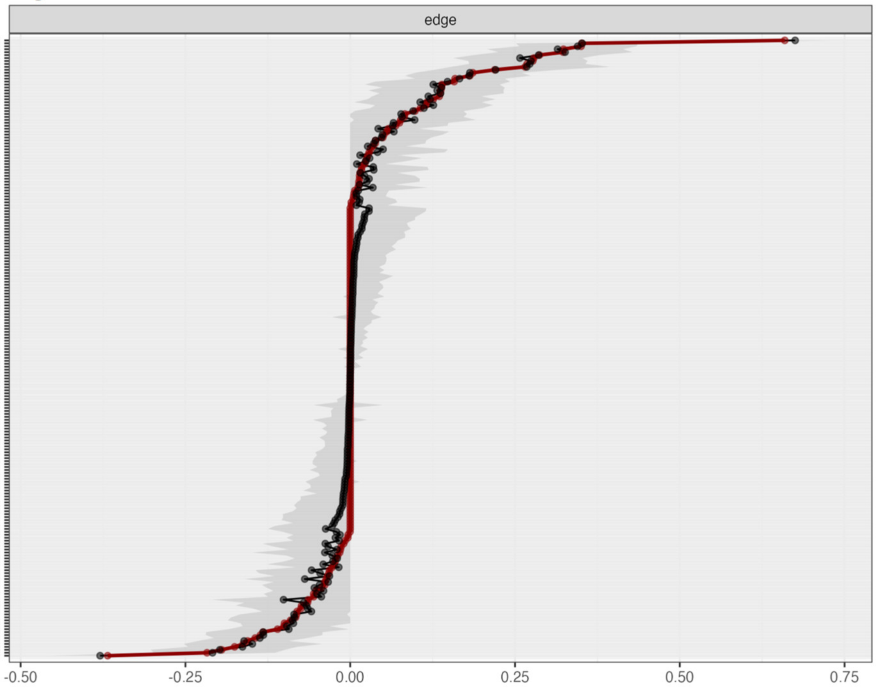

Supplement: Supplementary file 1 [file nutrients-16-03452-s001.zip › nutrients-3183619-supplementary.pdf]
